# Association Between CDH1 Downregulation and Lymphocyte Cell-Cycle Dysfunction in Alzheimer’s Disease and Mild Cognitive Impairment

**DOI:** 10.1007/s10571-026-01725-7

**Published:** 2026-04-15

**Authors:** Paloma Monllor, Begoña Lopez, Maria-Angeles Lloret, Jose-Luis Leon, Erika Lopez, Mari-Carmen Badia, Ana Lloret

**Affiliations:** 1 Department of Physiology, Faculty of Medicine, University of Valencia, INCLIVA, CIBERFES, Valencia, Spain; 2 Department of Internal Medicine, Hospital Universitario La Plana, Villareal, Castellon, Spain; 3 Department of Neurology, Hospital Universitario Doctor Peset, Valencia, Spain; 4 Department of Clinic Neurophysiology, Hospital Universitario y Politécnico La Fe, Valencia, Spain; 5 Ascires Biomedical Group, Department of Neuroradiology, Valencia, Spain; 6 Department of Neurology, Hospital Doctor Moliner, Bétera, Valencia Spain

**Keywords:** Cell cycle arrest, APC/C, Oxidative stress, Lymphocytes, Alzheimer’s disease

## Abstract

Alzheimer’s disease (AD) is characterized by the intracellular accumulation of amyloid-β and tau pathology, which disrupt multiple cellular regulatory pathways, including those involved in cell cycle control. Dysregulation of the anaphase-promoting complex/cyclosome (APC/C), particularly through altered expression of its coactivator CDH1, has been implicated in neuronal dysfunction in AD. Beyond the central nervous system, increasing evidence suggests that peripheral immune cells also exhibit functional alterations associated with the disease. In this study, we recruited patients with mild cognitive impairment (MCI) and AD, along with non-demented controls. We isolated T lymphocytes to assess mitogen-induced lymphocyte proliferation and associated molecular changes. We report that lymphocytes from MCI and AD patients show a reduced proliferative response compared to controls, accompanied by decreased CDH1 mRNA expression and altered expression of APC/C-related genes. These changes were associated with markers of oxidative stress and cellular stress pathways. Together, our findings identify an association between reduced CDH1 expression and impaired lymphocyte proliferation in AD and MCI, supporting the relevance of peripheral immune cells as candidates for systemic markers of disease-related cellular dysfunction.

**Graphical Abstract:**

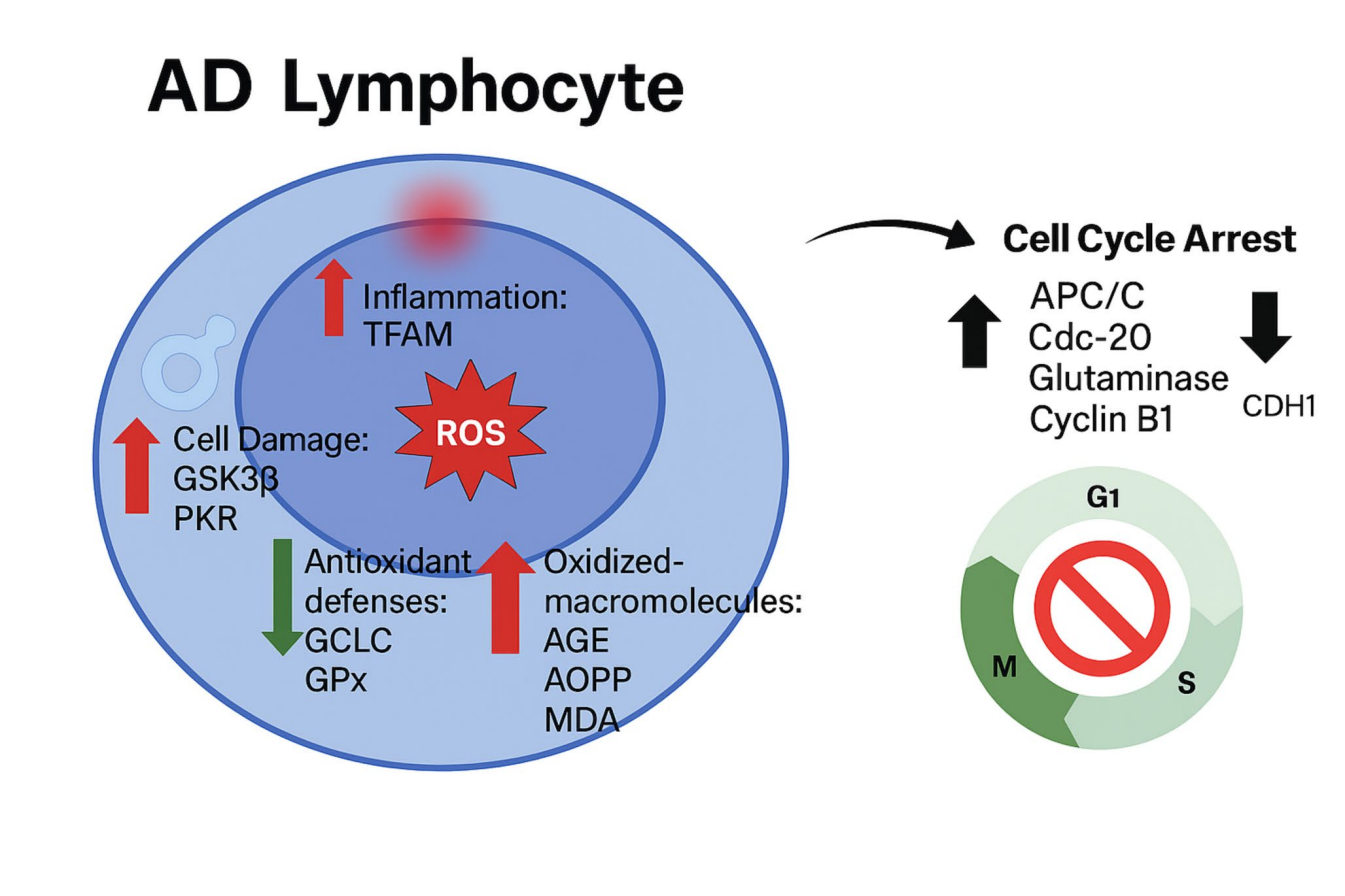

**Supplementary Information:**

The online version contains supplementary material available at 10.1007/s10571-026-01725-7.

## Introduction

Alzheimer’s disease (AD) is a neurodegenerative disorder characterized by progressive memory loss, cognitive decline, and behavioral changes. Early and accurate diagnosis is critical, prompting the search for reliable biomarkers. In fact, actual diagnosis criteria are based on imaging and/or biochemical biomarkers determined in cerebrospinal fluid (CSF) and/or plasma (Dubois et al. 2014, 2024). Plasma biomarkers are a good alternative due to the accessibility of obtaining samples and the broad variety of methods available nowadays. Despite this, it remains unclear which platform is best suited for measuring these biomarkers. While mass spectrometry methods and SIMOA (Single Molecule Array) platforms (Pais et al. 2023) are the most sensitive, there is no consensus on optimal cut-off values that can be applied across different samples to reliably distinguish between AD and non-AD cases.

Finding new peripheral biomarkers in cells could be another approach with high potential. Specifically, lymphocytes are a good model for studying brain disorders (Gladkevich et al. 2004), as they could reflect processes that take place in neurons. In addition, a good AD’s biomarker should reflect the biological basis of the disease’s continuum, and in this sense, lymphocytes could be an option for monitoring underlying pathological changes.

Beta-amyloid peptide, which accumulates in the AD patients’ brains, disrupts fundamental regulatory pathways, including those governing the cell cycle. Its progression is tightly controlled by regulatory proteins whose dysregulation can contribute to pathological conditions such as cancer or neurodegeneration as proved by several groups such as Plessir-Belair et al. (2025); Mathews et al. (2022).

A key regulator of cell cycle is the anaphase-promoting complex/cyclosome (APC/C), an E3 ubiquitin ligase that ensures timely protein degradation (Pagano 1997), driven forward the cell cycle progression. Its activity is modulated by the coactivators Cdh1 (E-cadherin) and Cdc20, which regulate different phases of the cell cycle by marking different proteins for degradation by the proteasome. In neurons, APC/C-Cdh1 is very active maintaining cells in G0 state, but it has been shown that Aβ42-induced depletion of APC/C-Cdh1 leads to an abnormal re-entry in the cell cycle.

In our previous experience, the inactivation of neuronal APC/C-Cdh1 in AD also causes accumulation of glutaminase, which in turn induce the increase of glutamate levels provoking overstimulation of NMDA receptors and excitotoxicity (Fuchsberger et al. 2016, 2017). Supporting this hypothesis, APP/PS1 transgenic mice supplemented with monosodium glutamate exhibited accelerated AD pathology, including increased Aβ42 and hyperphosphorylated tau accumulation and impaired synaptic function (Fuchsberger et al. 2019). These findings suggest that glutaminase plays a pivotal role in AD pathophysiology.

In addition to neuronal alterations, cell cycle dysregulation has also been identified in peripheral lymphocytes of AD patients. Peripheral blood T lymphocytes from AD patients, subjected to proliferation via a mitogen, expressed less CD69, an early proliferation marker, and its levels correlated inversely with the Mini-mental State Examination (MMSE) (Liston et al. 2022). Moreover, in immortalized lymphocytes from sporadic AD patients the level of the p21 protein was increased, which promotes G1-arrest, provoking a prolonged G1-phase in these cells (Bialopiotrowicz et al. 2011).

Recently, an age- and disease-associated decline in lymphocyte proliferation and decreased MMSE scores of MCI and AD patients were also observed (Vasantharekha et al. 2024). APC/C-Cdh1 plays a role in coordinating glycolysis and glutaminolysis with the transition to the S phase in human T lymphocytes as showed by Colombo et al. (2010). However, specific studies directly linking APC/C-Cdh1 activity in lymphocytes to AD are limited.

In this study we aim to assess if lymphocytes could reflect broader AD-related cellular dysfunction, offering a potential peripheral biomarker for early disease detection.

## Materials and Methods

### Subjects

Participants diagnosed with MCI (*n* = 34), patients diagnosed with AD (*n* = 18) and age-matched cognitively normal controls (*n* = 16) were recruited from the neurology service from the Hospital Clínico Universitario de Valencia according to the study’s inclusion and exclusion criteria. The exclusion criteria include the presence of neoplastic disease or immunosuppressive treatment for any reason, failure to sign the informed consent, and the absence of family members. Aged-matched non-demented controls were recruited from patients’ companions and the community. Inclusion criteria for control groups were having no history of cancer or other pro-inflammatory diseases and not suffering from any form of dementia.

Therefore, this is a prospective observational study. All participants went through a medical interview where the MMSE was performed to evaluate cognitive status. Afterwards, 20 ml of blood was collected from the antecubital vein of each participant. Participation in the study was voluntary, and informed consent was obtained from all individual participants or, if a subject was not able to sign the consent, it was signed by a family member responsible for them. This study was conducted in accordance with the Helsinki declaration, UNESCO’s Universal Declaration on Bioethics and Human Rights, the International Ethical Guidelines for Biomedical Research involving Human Subjects from the Council for International Organizations of Medical Sciences, the Council of Europe’s Convention on Human Rights and Biomedicine and the European Charter of Fundamental Rights.

Furthermore, it was conducted in accordance with the Spanish legislation regarding personal data protection, the Spanish Bioethics Law and was approved by the Ethics Committee of the Hospital Clinico Universitario de Valencia. Demographic and clinical data for each group is presented in the results section.

### Lymphocyte Isolation and Proliferation Induction

Blood samples were obtained by venipuncture from the cubital vein and collected into BD Vacutainer^®^ CPT™ Mononuclear Cell Preparation Tubes containing Sodium Citrate (#362761). Following centrifugation at 1,800 x g for 35 min at 22 °C, mononuclear cells were harvested. Plasma was also collected, aliquoted, and stored at -80 °C for potential future use. Mononuclear cells were washed once with RPMI culture media (Sigma-Aldrich). Afterwards, the cells were incubated for 3 h at 37 °C in RPMI supplemented with 5% fetal bovine serum (Capricorn scientific) and 1% penicillin/streptomycin. Floating lymphocytes were carefully harvested and counted using a cell counter (TC10 automated cell counter; Bio-Rad).

Lymphocytes were then seeded at a density of one million cells per Petri dish in fresh culture medium supplemented with 2.5% phytohemagglutinin (PHA, Gibco) and incubated for 48 h. The 2.5% PHA concentration represented an optimized concentration determined through preliminary testing. Following the 48-hour incubation, cells were collected by centrifugation at 1500 G for 10 min, the resultant cell pellet was then resuspended and washed with PBS, using a second centrifugation at 1500 G for 10 min. The objective was to obtain a purified T lymphocyte population, which was then stored at -80 °C for downstream analysis.

During that analysis all laboratory personnel and researchers involved in sample processing and data acquisition were blinded to the diagnostic group (MCI, AD, or control) of the samples. The key linking the codes to the diagnostic groups was only revealed after all data collection was finalized.

### PCR Analysis

RNA was isolated from lymphocytes using the PARIS Protein and RNA isolation kit (Ambion, #AM1921) according to the manufacturer’s instructions. For the reverse transcription (RT) reaction, 1 mg of purified RNA was transcribed using random hexamers with the cDNA Archive High-Capacity kit (Applied Biosystems). Reverse transcription conditions were an initial incubation at 25 °C for 10 min, followed by cDNA synthesis reaction at 37 °C for 120 min and a final inactivation step of 5 min at 95 °C. The measurement of mRNA levels was determined by quantitative PCR with the ABI Prism 7900 HT Fast Real-Time PCR System (Applied Biosystems). The specific primers used were obtained from Qiagen (Applied Biosystems). The PCR conditions were 10 min at 95 °C to activate the enzyme, followed by 40 cycles of 15 sat 95 °C and 1 min at 60 °C. The expression levels of glyceraldehyde-3-phosphate dehydrogenase (GAPDH) were measured in all samples with the aim of normalizing the expression of each gene, RNA quality, and efficiency of RT. The primers were from TaqMan Gene Expression Assays *ANAPC7* (Hs01067023_m1); *CDH1* (Hs01023895_m1), *CCNB1* (Hs01565448_g1), *CDC20* (Hs00961704_g1), *GLS1* (Hs01014028_m1), *GCLC* (Hs00155249_m1), *GCLM* (Hs00157694_m1), *GPX1* (Hs00829989_gH), *GSK3B* (Hs00275656_m1), *PKR* (Hs00169345_m1),, *GAPDH* Hs02786624_g1. Each sample was tested in triplicate, and the expression was calculated according to the method 2^−ΔΔCt^.

### ELISA Determinations

CSF levels of P-tau_181_, and Aβ_42_ were determined by solid-phase enzyme immunoassay (Innogenetics, Ghent, Belgium; references: INX74378, INX27352, respectively). Pathological cutoff values were established according to the laboratory’s internal validation and manufacturer guidelines: **< 500 pg/mL for Aβ42** or **> 61 pg/mL for p-Tau181.** Plasma levels of were determined by the same technique following the manufacturer`s recommendations in all the tests. All samples were assayed in duplicate.

### Statistical Analysis

A *priori* sample size calculation was performed to determine the appropriate sample size. Based on previous experiments in our group regarding plasma biomarker determinations, and assuming a statistical power of 0.88 and a significance level of 0.05, the target sample size was established at 30 participants per group.

Following this, participants diagnosed with MCI (*n* = 34), patients diagnosed with AD (*n* = 18) and age-matched cognitively normal controls (*n* = 16) were recruited from the neurology service from the Hospital Clínico Universitario de Valencia according to the study’s inclusion and exclusion criteria. The final sample recruited was smaller than the target size due to practical recruitment constraints during the study period.

First, a descriptive analysis of the sample was performed, grouping variables into the following categories: demographic variables, personal history, family history, clinical variables, functional status, neuropsychological assessment, and laboratory variables. Absolute and relative frequencies were calculated for categorical variables. Continuous variables were assessed using measures of central tendency and dispersion. Normality of data distribution was assessed to ensure appropriate methodology. The Kolmogorov-Smirnov test was used to assess normality, with a standard 95% confidence level (p-value > 0.05) indicating normal distribution in most groups and variables.

For normally distributed samples, the mean and standard deviation (mean ± standard deviation) were used. For non-normally distributed samples, the median was used as the measure of central tendency, and the mode was used as the measure of dispersion.

Differences between groups were evaluated using hypothesis testing. For comparing means of more than two categories of continuous variable, one-way ANOVA was used, and the Kruskal-Wallis Test was used for comparing more than two groups in non-normally distributed variables. The assumption of homogeneity of variances was assessed using Levene’s Test. If variances were homogeneous (*p* > .05), Bonferroni post-hoc analysis was used to identify groups with significant differences. If the assumption was violated (*p* < .05), a Welch’s ANOVA with the Games-Howell post-hoc test was used. All tests were performed using SPSS version 24 (IBM).

In supplemental material a detailed explanation of each result can be found.

## Results

### Participant Characteristics

The demographic and clinical characteristics of the study participants are detailed in Table 1. The mean age of the cohort was 69 years. There was a trend towards a higher proportion of female participants in both the control (68%) and Alzheimer’s disease (AD) groups (66%), however, this difference did not reach statistical significance. MMSE scores for both groups are also provided in Table 1.

**Table 1:** Demographic characteristics for the participants

Participants (*n*)	Control	MCI	AD	*p*
	16	34	18	-
Baseline age (mean + SD)	67,6 ± 8,8	68,6 ± 6,3	69,2 ± 6,5	0.326
Female n (%)	11 (68%)	15 (44%)	12 (66%)	0.244
MMSE	28 ± 1	25 ± 3	21 ± 5	0.000
Abbreviations: MCI: Mild Cognitive Impairment, AD: Alzheimer’s disease; MMSE, Mini Mental State Examination				

**Fig. 1:**
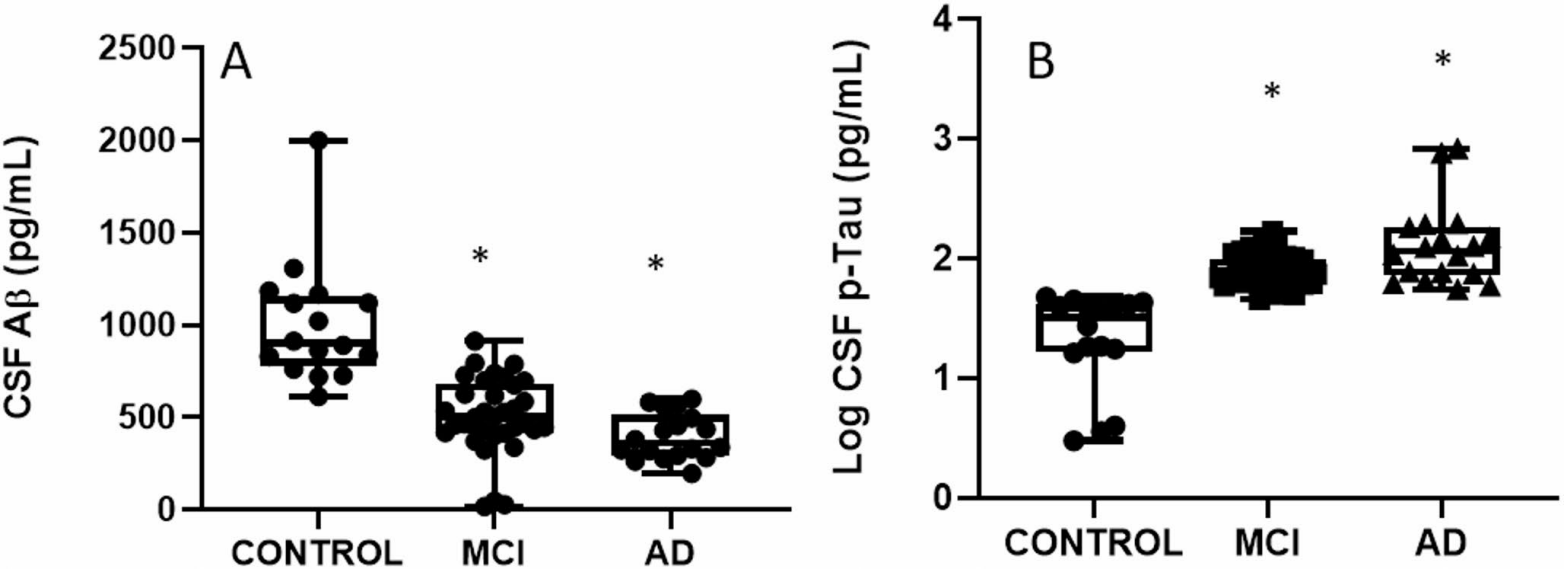
CSF biomarkers AB **(A**) and log p-Tau (**B**) are significantly altered in MCI and AD patients. The horizontal line represents the median, the box represents the interquartile range, and whiskers represent the minimum and maximum values. *p < .05 vs. Control

Analysis of CSF revealed significant alterations in AD biomarkers, Aβ42 and pTau181 were used as diagnostic criteria using the ATN guidelines (Jack et al. 2018). As depicted in Fig. 1, AD patients exhibited significantly decreased levels of Aβ42 in comparison to control subjects (Fig. 1A). Conversely, p-tau levels were significantly elevated in the CSF of both MCI and AD patients compared to controls (Fig. 1B). These findings are consistent with the known biochemical hallmarks of AD pathogenesis (Dubois et al. 2014).

### Lymphocyte Proliferation is Impaired in MCI and AD Patients compared to controls

**Fig. 2:**
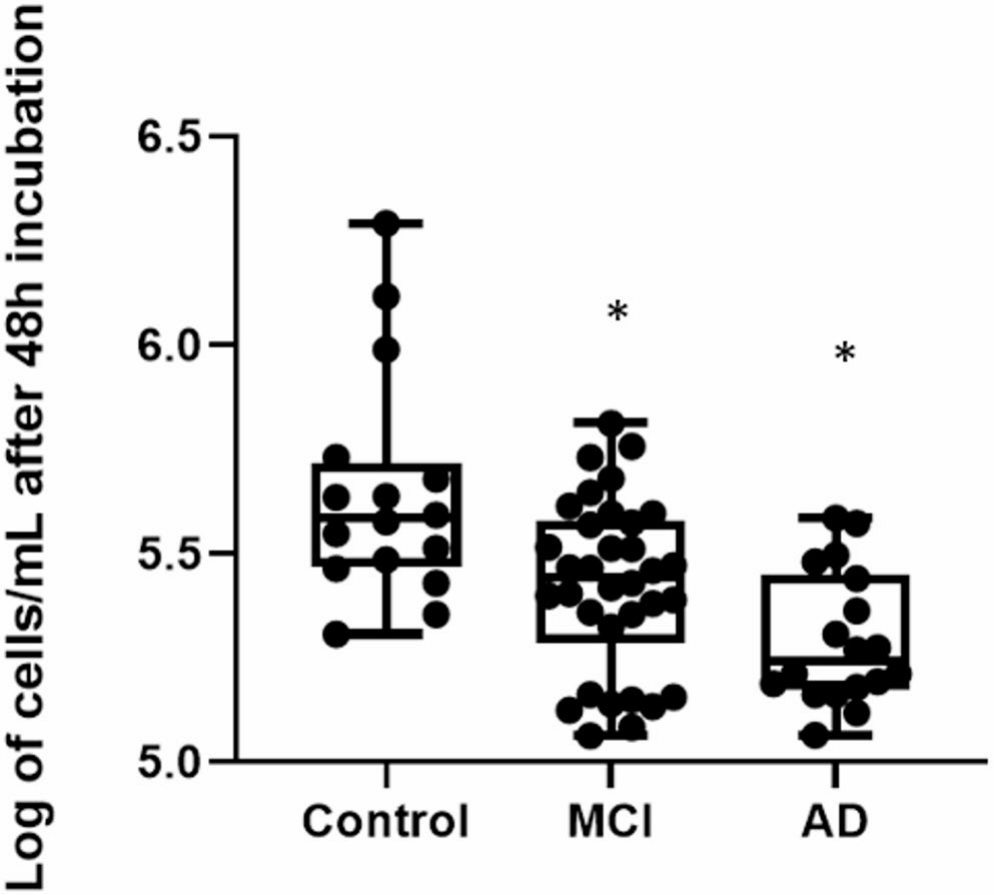
Lymphocyte proliferation is significantly impaired in both MCI and AD patients. Box-and-dot plots show the logarithm of lymphocytes proliferation from Control, MCI, and AD subjects after 48 h of culture. The number of cells/mL is significantly reduced in the MCI and AD groups compared to the control group. The horizontal line represents the median, the box represents the interquartile range, and whiskers represent the minimum and maximum values. *p < .05 vs. Control

Building on existing evidence suggesting peripheral immune dysfunction in Alzheimer’s disease (Butovsky et al. 2025), we first sought to determine if basic lymphocyte function, such as proliferation, was affected in our patient cohorts. The assessment of lymphocyte proliferation following 48 h of culture revealed a significant reduction in cell number in both MCI and AD patients compared to the control group (Fig. 2).

### The APC/C-Cdh1 Cell Cycle Machinery is Dysregulated in MCI and AD compared to controls

Given these observed deficits in lymphocyte proliferation, we next hypothesized that their underlying regulatory machinery might be dysregulated. We therefore investigated APC/C, the master regulator of the cell cycle, and its cofactors Cdh1 and Cdc20, which are essential for cell cycle progression (Kramer et al. 2000).

**Fig. 3:**
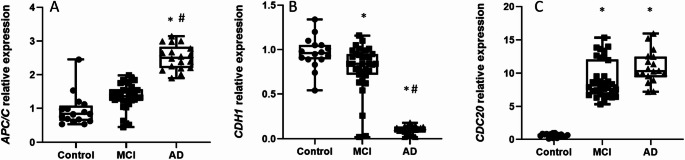
The APC/C complex is upregulated while its key cofactor Cdh1 is downregulated in MCI and AD patient lymphocytes. Box-and-dot plots show the relative expression of (A) APC/C, (B) Cdh1, and (C) Cdc20 measured by qPCR. APC/C levels are significantly increased in the AD group compared to controls. In contrast, Cdh1 expression is significantly reduced in the AD group. Cdc20 expression is significantly increased in both MCI and AD groups compared to controls. The horizontal line represents the median, the box represents the interquartile range, and whiskers represent the minimum and maximum values. *p < .05 vs. Control; *p* < .05 vs. MCI. Data are normalized to [GAPDH] and expressed relative to the Control group (set to 1)

We found that the expression of APC/C (*ANAPC7*) was significantly higher in AD patients than in control and MCI groups (Fig. 3A). However, we observed a significant reduction in its cofactor Cdh1 (*CDH1*) (Fig. 3B) in both MCI and AD groups when compared with controls. Moreover, this reduction of Cdh1 levels was accompanied by a significant increase in Cdc20 (*CDC20*) expression (Fig. 3C). This combined dysregulation of the APC/C-Cdh1 and APC/C-Cdc20 balance strongly suggests a disruption in cell cycle checkpoints control, which can explain the reduced lymphocyte proliferation we observed.

### Cdh1 Downregulation is Associated with the Accumulation of its Target Substrates

Given our key finding that *CDH1* was downregulated, we next explored whether this change was associated with alterations in two of its main target proteins. Specifically, we examined the levels of glutaminase, a key enzyme in the glutamate/glutamine cycle, and cyclin B1, a regulator of cell cycle progression.

**Fig. 4:**
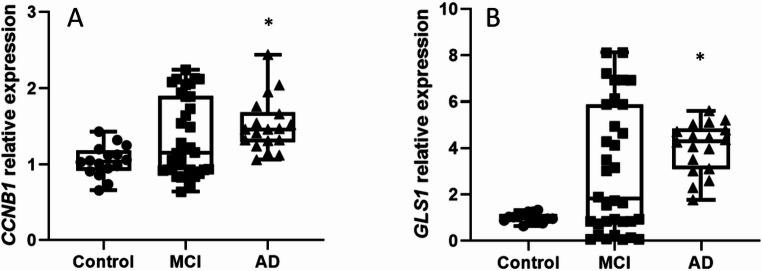
Downregulation of Cdh1 is associated with the significant accumulation of its substrates, Cyclin B1 and Glutaminase. Box-and-dot plots show the relative mRNA expression of (A) Cyclin B1 and (B) Glutaminase. Both Cyclin B1 and Glutaminase levels are significantly increased in the AD group compared to controls, consistent with impaired degradation by the APC/C-Cdh1 complex. The horizontal line represents the median, the box represents the interquartile range, and whiskers represent the minimum and maximum values. *p < .05 vs. Control. Data are normalized to [GAPDH] and expressed relative to the Control group (set to 1)

As hypothesized, Fig. 4 reveals that both cyclin B1 (*CCNB1*) (Fig. 4A) and glutaminase (GLS1) (Fig. 4B) expressions are significantly increased in the AD group compared to the control group. This suggests that the observed dysregulation of the APC/C-Cdh1 pathway leads to the accumulation of its target substrates, which may in turn contribute to altered glutamate metabolism and aberrant cell cycle progression.

### Patients with AD Exhibit Increased Systemic Oxidative Damage

Our findings so far point to a cascade of dysfunction: impaired lymphocyte proliferation and dysregulated cell cycle machinery. Since both cell cycle arrest and glutamate-induced excitotoxicity are known to be primary drivers of oxidative damage, we next sought to determine if these alterations were associated with a systemic oxidative stress profile in our patients.

**Fig. 5:**
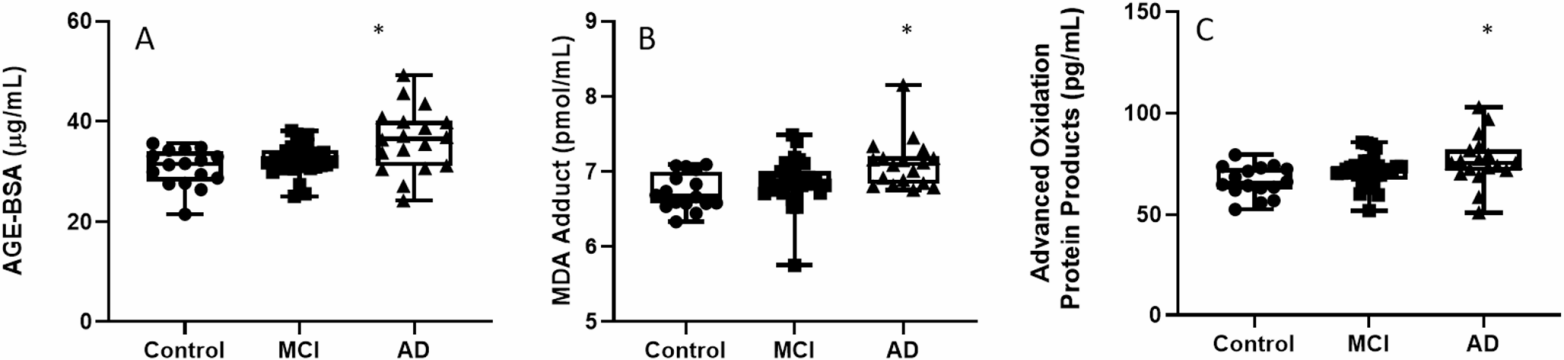
Systemic markers of oxidative damage are significantly increased in AD patients. Box-and-dot plots show the plasma levels of **A** AGE-BSA, **B** MDA Adduct, and **C** Advanced Oxidation Protein Products (AOPP). All three markers of oxidative stress are significantly elevated in the AD group compared to controls. No significant differences were observed between the control and MCI groups for any marker. The horizontal line represents the median, the box represents the interquartile range, and whiskers represent the minimum and maximum values. *p < .05 vs. Control

To this end, plasma markers of oxidative damage to different cellular macromolecules like advanced glycation end-products (AGE), malondialdehyde (MDA), and advanced oxidation protein products (AOPP) were measured. As depicted in Fig. 5, all three oxidative stress biomarkers were significantly increased in AD subjects compared to controls.

### The Glutathione-Based Antioxidant Defense System is Compromised in Patients

This significant increase in systemic oxidative damage led us to investigate the body’s primary antioxidant defense system. A possible imbalance between oxidation and antioxidation could explain the high levels of damage observed. We therefore measured the expression of key enzymes in the glutathione metabolism pathway, the body’s most abundant intracellular antioxidant.

**Fig. 6:**
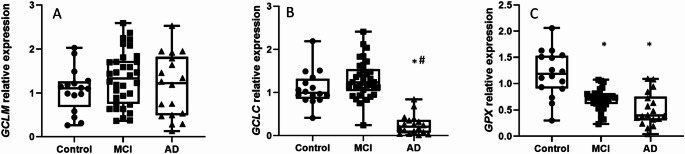
The antioxidant defense system is compromised in patients, characterized by the downregulation of *GCLC* and *GPX*. Box-and-dot plots show the relative mRNA expression of the glutathione metabolism enzymes **A** *GCLM*, **B** *GCLC*, and **C** *GPX*. While *GCLM* levels remain unchanged across groups, expression of the catalytic subunit *GCLC* is significantly downregulated in the AD group compared to controls. Furthermore, *GPX* expression is significantly reduced in both MCI and AD groups compared to controls. The horizontal line represents the median, the box represents the interquartile range, and whiskers represent the minimum and maximum values. *p < .05 vs. Control; *p* < .05 vs. MCI. Data are normalized to [GAPDH] and expressed relative to the Control group (set to 1)

We measured the mRNA levels of the glutamate-cysteine ligase (GCL) catalytic (GCLC) and modifier (GCLM) subunits, and glutathione peroxidase (GPX). As depicted in Fig. 6, no statistically significant differences were observed in the mRNA levels of GCLM (Fig. 6A). However, GCLC mRNA levels were significantly decreased in AD patients compared to control subjects (Fig. 6B), and GPX mRNA levels decreased in both MCI and AD groups compared to non-demented controls (Fig. 6C).

### Cellular Stress Markers are Elevated in MCI and AD

Our findings of significant oxidative damage (high MDA/AOPP) coupled with a compromised antioxidant defense system (low *GCLC/GPX*) point to a state of profound cellular stress. As a final step, we sought to determine if this stress profile was associated with alterations in key downstream kinases and mitochondrial markers deeply implicated in AD. We therefore assessed the expression of the stress-response kinases GSK3β (*GSK3B)* and EIF2AK2 (*PKR*), as well as the mitochondrial transcription factor TFAM, to gain insight into mitochondrial function.

**Fig. 7:**
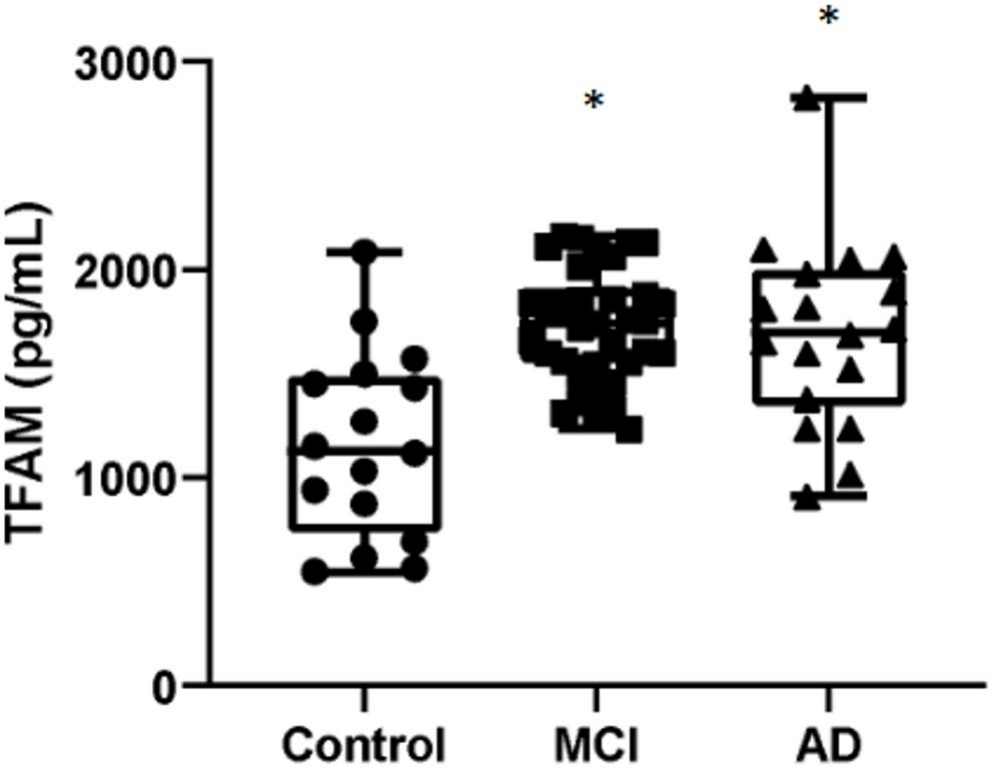
Mitochondrial transcription factor TFAM is significantly elevated in both MCI and AD patients. Box-and-dot plots show the plasma levels of TFAM. TFAM concentrations are significantly increased in both the MCI and AD groups compared to healthy controls, suggesting an early and sustained mitochondrial stress response. No significant differences were observed between the MCI and AD groups. The horizontal line represents the median, the box represents the interquartile range, and whiskers represent the minimum and maximum values. *p < .05 vs. Control

**Fig. 8:**
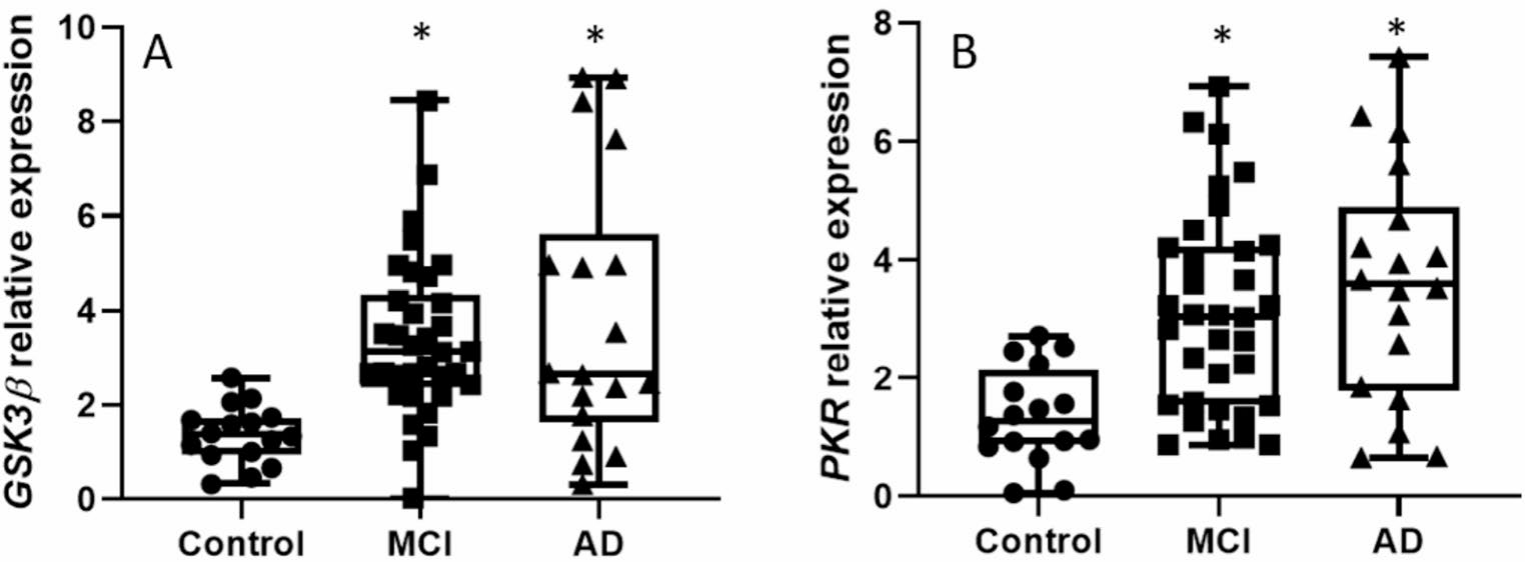
Stress-response kinases *GSK3β* and *PKR* are significantly upregulated in MCI and AD lymphocytes. Box-and-dot plots show the relative mRNA expression of **A** *GSK3β* and **B** PKR (*EIF2AK2*). Both kinases show a highly significant increase in expression in the MCI and AD groups compared to healthy controls, indicating a robust cellular stress response. No significant differences were observed between the MCI and AD groups for either kinase. The horizontal line represents the median, the box represents the interquartile range, and whiskers represent the minimum and maximum values. *p < .05 vs. Control. Data are normalized to [GAPDH] and expressed relative to the Control group (set to 1)

The results showed that plasma TFAM levels were elevated in both the MCI and AD groups compared to controls (Fig. 7). Additionally, we found that both GSK3β and PKR were significantly increased in both the MCI and AD groups compared to controls (Fig. 8A and B).

## Discussion

### Alzheimer’s Disease and T Lymphocyte Cell Cycle

Lymphocytes are suitable models reflecting CNS cellular dysfunction in AD due to their cellular and biochemical similarities to neurons and their accessibility (Kerage et al. 2019). Earlier studies such as those conducted by Kalman et al. (2005) and Maes et al. (2007) reported that lymphocytes in AD pathology display dysregulated signaling pathways, altered lipid metabolism, mitochondrial bioenergetic dysfunction, and reduced cell survival. Recent focus on immune involvement highlights that peripheral T cells can infiltrate the CNS (Mrdjen et al. 2018) locating themselves in regions typically associated with AD neuropathology, such as the hippocampus, corpus callosum, cingulate and cortex (Togo et al. 2022; Batterman et al. 2021). The importance of studies regarding the state of the immune system in AD is of particular interest due to the latest approved treatments based on immunotherapies against beta-amyloid peptide.

In this study, we observed that T lymphocytes from patients with MCI and AD exhibited reduced proliferative responses compared to controls. Dysregulation of the cell cycle has been reported in peripheral cells of AD patients, including lymphocytes (Stieler et al. 2001; Bialopiotrowicz et al. 2011; Lapresa et al. 2022), but the molecular basis has not been studied deeply. Specifically, the G1/S transition control mechanism is compromised in lymphocytes from AD patients (Song et al. 2012). Our results suggest that the previously observed G1/S transition failure is likely linked to dysfunction of the APC/C-Cdh1 complex. We found significantly reduced Cdh1 levels in proliferating lymphocytes from MCI and AD patients compared to non-demented controls. We previously reported that exposure to Aβ42 peptides in cultured neurons and hippocampal neurons from an AD murine model also led to decreased Cdh1 levels (Fucheberger et al. 2016). We now demonstrate similar behavior in lymphocytes from MCI and AD patients. Furthermore, we observed significantly increased expression of APC/C and its cofactor Cdc20 in MCI and AD. We hypothesize that without the necessary machinery to pass through G1, this might result in an aberrant cell cycle.

Although CDH1 is a well-established negative regulator of CDC20 at the protein level through APC/C-mediated ubiquitination and proteasomal degradation, our qPCR data reveal an apparent dissociation between transcriptional and post-translational regulation. Specifically, reduced CDH1 mRNA levels are accompanied by an increase in CDC20 mRNA expression. This finding is consistent with previous reports indicating that CDC20 transcription is primarily controlled by cell-cycle–dependent transcription factors, such as E2F and FOXM1, rather than directly by APC/C–CDH1 activity (Emanuele et al. 2020; Xie et al. 2015). Thus, the observed upregulation of CDC20 mRNA likely reflects a compensatory transcriptional response to impaired APC/C–CDH1 function and altered cell-cycle progression, rather than a direct regulatory effect of CDH1 on CDC20 gene expression.

APC/C-Cdc20 primarily functions during mitosis, regulating metaphase-to-anaphase transition and mitotic exit, whereas APC/C-Cdh1 is active in late mitotic exit and early G1 phase (Dragoi et a. 2024), also playing a key role in DNA damage response and repair. Cdh1 is crucial for maintaining genomic integrity, as its dysregulation has been linked to cytokinesis defects, DNA re-replication, and centrosome abnormalities (Wäsch et al. 2010). Therefore, Cdh1 downregulation could serve as an early peripheral marker associated with AD-related cellular dysfunction and could explain the observed proliferation deficits in lymphocytes.

Consistently, we found increased levels of APC/C-Cdh1 targets, including glutaminase1 and cyclin B1 in AD patients. This accumulation is consistent with impaired degradation due to reduced Cdh1 expression and APC/C dysfunction. However, in MCI patients, these differences were not statistically significant. It should also be kept in mind that MCI patients could be an intermediate stage between non-dementia and established dementia, and the results of many studies are not uniform (Petersen et al. 2014). In fact, many MCI patients do not progress to dementia and remain in this stage for long periods of time (Lee 2023).

The increased levels of cyclin B1 observed in AD patients are particularly relevant, as this protein is involved in cell mitotic entry (Zheng et al. 2024). Therefore, its accumulation in AD lymphocytes could contribute to aberrant cell cycle progression and impaired proliferation. Elevated cyclin B1 levels in neurons have also been associated with mitochondrial dysfunction and oxidative stress, as well as cell death induction (de Tudela et al. 2015).

Together, our findings highlight the dysregulation of APC/C-Cdh1 in AD pathology and suggest that further investigation into its role in lymphocyte dysfunction may provide novel insights into disease mechanisms and potential biomarkers for early detection.

### Glutamate Dysregulation in AD

We have observed an upregulation of GLS1 in MCI and AD that aligns with decreased APC/C-Cdh1 ubiquitination function. We previously showed that Aβ42 or excess glutamate induces APC/C-Cdh1 depletion, leading to GLS1 accumulation and neurotoxicity (Fuchsberger et al. 2016). In lymphocytes, glutamine fuels proliferation. While altered glutamine metabolism is well-studied in cancer (Dai et al. 2020), its role in AD is emerging. Recent bioinformatic studies identified altered glutamine metabolism genes in AD regulating immune-inflammatory responses (Wu et al. 2023), making this a promising research avenue.

### Oxidative Stress Increased in Lymphocytes from AD Patients

We found a significant decrease in GCLC in AD patients, suggesting impaired antioxidant defenses. Given that GSH synthesis is needed to increase during lymphocyte proliferation (Muri and Kopf 2021), this deficit could be a consequence of increased oxidative stress (that would deplete GSH levels) and could cause reduced cell cycle activity in AD as suggested early by Fernandez-Checa et al. (2020).

Our results indicate a decrease in the antioxidant enzyme GPX in both MCI and AD patients compared to controls, as well as a reduction in GCLC levels in AD compared to both controls and MCI. Furthermore, we observed increased plasma levels of MDA and AGEs, suggesting a state of oxidative stress in MCI and AD patients. These findings are consistent with previous studies that have described systemic oxidative stress in AD (Lloret et al. 2009).

Several studies have associated AD with the reduction of cellular antioxidant defenses (Nantachai et al. 2022) including GPX, in fact, there is a study that relates this depletion to cognitive decline (Baldeiras et al. 2010). In AD patients’ lymphocytes, there is a reported lower GSH levels alongside increased oxidized GSSG levels (Butterfield and Boyd-Kimball 2018), supporting the hypothesis that a dysregulated redox balance plays a role in neurodegeneration. Additionally, decreased serum levels of GPX, along with elevated MDA, have been previously observed in both MCI and AD patients compared to controls (Torres et al. 2011). Our results align with these findings regarding MDA and GPX expression. In fact, peripheral oxidative damage to lipids and proteins has been widely reported in neurodegenerative diseases (Perluigi et al. 2024), with some studies suggesting that lipid peroxidation markers could mediate the progression from MCI to AD (Butterfield et al. 2010).

In addition to its role in neurodegeneration, oxidative stress has been shown to interfere with cell cycle regulation (Shackelford et al. 2000; Plessis-Belair et al. 2025). Peroxides and ROS can impair cell cycle checkpoints at different stages, particularly in G1 and G2 to M progression by reducing cyclin B activity (Menon and Goswami 2007; Guo et al. 2010; Patterson et al. 2019). This arrest is often mediated by oxidative damage to key regulatory proteins, including cyclins and CDKs. This stress-induced arrest can lead either to cell cycle blockade or to cell death, depending on the cellular context. In our study, we observed altered expressions of key cell cycle regulators such as Cdh1 and its substrates, suggesting that oxidative stress might compromise cell cycle progression in peripheral lymphocytes from AD patients. Given these observations, oxidative stress markers such as MDA and carbonylated proteins have been suggested as potential peripheral biomarkers for early MCI diagnosis, while reduced GSH levels might serve as indicators of progression to AD (Perluigi et al. 2024; Collin et al. 2018).

### Increased Expression of Stress-Related Genes in AD

We observed increased gene expression of GSK3β, PKR and TFAM. These proteins are deeply involved in AD pathophysiology, especially in the formation of hyperphosphorylated tau and in mitochondrial signaling. Interestingly, previous studies from our group found increased GSK3β and PKR levels even in cognitively healthy individuals carrying the ApoE4 genotype, reinforcing the idea that these alterations precede clinical symptoms (Badia et al. 2012).

The persistent activation of GSK3β and PKR, as observed in AD, contributes to tau pathology and neuronal degeneration as widely supported by several studies (Badia et al. 2012; Paquet et al. 2012; Hernandez et al. 2012; Jin et al. 2015). In addition to being kinases that phosphorylate tau, GSK3β and PKR are two important regulators of the cellular damage control response such as, cell cycle arrest, cell death and inflammation (Piazzi et al. 2020; Cortés-Vieyra et al. 2021).

Indeed, in AD, PKR has been shown to induce the overexpression of GSK3β by inducing tau hyperphosphorylation. For example, a PKR inhibitor reduced the levels of GSK3β and P-tau (Hugon et al. 2017). Interestingly, in lung cancer cells, GSK3β has been shown to increase glutaminase expression (Momcilovic et al. 2018). We have shown elevated expression levels of both enzymes in our samples although the mechanism in AD is still to be clarified.

TFAM is a mitochondrial transcription factor that is known to activate microglia in the brain and induce the synthesis of proinflammatory interleukins such as IL1beta. While GSK3β typically inhibits TFAM expression (Undi et al. 2017), in our patients, both proteins were elevated, possibly reflecting a compensatory mechanism or an inflammatory response to the accumulation of toxic proteins such as Aβ42 and hyperphosphorylated tau.

However, when a cell is under stress, as may be the case in AD lymphocytes, the cells arrest their cell cycle to adapt to the new environment. Cells adjust the length of cell cycle arrest for each individual stressor, but how they do this is not well understood. Altogether, our findings highlight the contribution of systemic oxidative stress and abnormal stress signaling pathways to the pathogenesis and progression of AD, supporting the hypothesis that AD may arise from a chronic systemic stress condition, rather than being solely a consequence of localized neurodegeneration.

### Limitations of the Study

Our study presents several limitations. First, the sample size—particularly in the AD group—was smaller than planned, rendering the study underpowered and requiring cautious interpretation of the findings. Second, its monocentric, cross-sectional design limits generalizability and prevents causal inferences, necessitating future longitudinal studies. Third, our analyses primarily rely on mRNA expression, which may not fully reflect protein levels or functional activity, especially for post-translationally regulated complexes like APC/C. Fourth, further phenotypic characterization (e.g., CD3 + flow cytometry) would better define the isolated cell populations. Finally, while peripheral lymphocytes are valuable models for studying systemic alterations, they reflect peripheral dysfunction rather than acting as direct surrogates for central nervous system pathology.

## Supplementary Information

Below is the link to the electronic supplementary material.

Supplementary Material 1

## Data Availability

The datasets generated during and/or analyzed during the current study are not publicly available due but are available from the corresponding author on reasonable request.
