# Supplementary material for: Association Between CDH1 Downregulation and Lymphocyte Cell-Cycle Dysfunction in Alzheimer’s Disease and Mild Cognitive Impairment
Source: Cell Mol Neurobiol. 2026 Apr 15;46:93. doi: 10.1007/s10571-026-01725-7 (PMC13199537; doi:10.1007/s10571-026-01725-7)
Supplement: Supplementary file 1 — Supplementary Material 1 [file 10571_2026_1725_MOESM1_ESM.pdf]

TABLE:

Grupo	Variable	Normality K-S: D(df)	Normality K-S: p-value	Homogeneity Levene's: F(df1, df2)	Homogeneity Levene's: p-value	Final Test Used
control	AB	D(16): 0,172	0,200	F(2, 65) = 3.29	0,044	Welch's ANOVA
MCI		D(34): 0,128	0,174			
AD		D(18): 0,178	0,137			
control	ptau	D(16): 0,222	0,034	N/A	N/A	KRUSKALL WALLIS TEST
MCI		D(34): 0,180	0,007			
AD		D(18): 0,376	0,000			
control	lymphocytes	D(16): 0,326	0,000	N/A	N/A	KRUSKALL WALLIS TEST
MCI		D(34): 0,115	0,200			
AD		D(18) 0,172	0,170			
control	APC	D(16): 0,229	0,024	N/A	N/A	KRUSKALL WALLIS TEST
MCI		D(34): 0,173	0,011			
AD		D(18): 0,121	0,200			
control	Cdh1	D(16): 0,160	0,200	N/A	N/A	KRUSKALL WALLIS TEST
MCI		D(34): 0,174	0,011			
AD		D(18): 0,151	0,200			
control	cdc20	D(16): 0,137	0,200	N/A	N/A	KRUSKALL WALLIS TEST
MCI		D(34): 0,200	0,001			
AD		D(18): 0,146	0,200			
control	glutaminasa	D(16): 0,143	0,200	N/A	N/A	KRUSKALL WALLIS TEST
MCI		D(34): 0,192	0,030			
AD		D(18): 0,148	0,200			
control	cyclin B1	D(16): 0,093	0,200	N/A	N/A	KRUSKALL WALLIS TEST
MCI		D(34): 0,181	0,007			
AD		D(18): 0,191	0,080			

control	plasma glutamate	D(16): 0,127	0,200	N/A	N/A	KRUSKALL WALLIS TEST
MCI		D(34): 0,102	0,200			
AD		D(18): 0,180	0,130			
control	plasma glutamine	D(16): 0,145	0,200	F(2, 65) = 2,219	0,117	ANOVA
MCI		D(34): 0,075	0,200			
AD		D(18): 0,078	0,200			
control	csf_glutamate	D(16): 0,139	0,200	N/A	N/A	KRUSKALL WALLIS TEST
MCI		D(34): 0,187	0,004			
AD		D(18): 0,123	0,200			
control	csf_glutamine	D(16): 0,110	0,200	F(2, 65) = 2,739	0,072	ANOVA
MCI		D(34): 0,143	0,078			
AD		D(18): 0,163	0,200			
control	csf_GABA	D(16): 0,149	0,200	F(2, 65) = 24,664	0,000	Welch's ANOVA
MCI		D(34): 0,123	0,200			
AD		D(18): 0,132	0,200			
control	GCLC	D(16): 0,178	0,190	F(2, 65) = 2,487	0,091	ANOVA
MCI		D(34): 0,114	0,200			
AD		D(18): 0,152	0,200			
control	GCLM	D(16): 0,162	0,200	F(2, 65) = 2,397	0,099	ANOVA
MCI		D(34): 0,085	0,200			
AD		D(18): 0,156	0,200			
control	GPx	D(16): 0,121	0,200	F(2, 65) = 7,798	0,001	Welch's ANOVA
MCI		D(34): 0,137	0,105			
AD		D(18): 0,190	0,086			
control	AGE	D(16): 0,130	0,200	F(2, 65) = 5,793	0,005	Welch's ANOVA
MCI		D(34): 0,140	0,089			
AD		D(18): 0,079	0,200			

control	MDA	D(16): 0,155	0,200	F(2, 65) = 1,118	0,333	ANOVA
MCI		D(34): 0,144	0,073			
AD		D(18): 0,193	0,076			
control	AOPP	D(16): 0,127	0,200	F(2, 65) = 1,597	.210	ANOVA
MCI		D(34): 0,137	0,103			
AD		D(18): 0,152	0,200			
control	PKR	D(16): 0,125	0,200	F(2, 65) = 4,096	0,021	Welch's ANOVA
MCI		D(34): 0,114	0,200			
AD		D(18): 0,101	0,200			
control	GSK3B	D(16): 0,087	0,200	N/A	N/A	KRUSKALL WALLIS TEST
MCI		D(34): 0,121	0,200			
AD		D(18): 0,217	0,025			
control	TFAM	D(16): 0,101	0,200	F(2, 65) = 2,938	0,06	ANOVA
MCI		D(34): 0,094	0,200			
AD		D(18): 0,130	0,200			

RESULTS:

- **CSF_AB:** For CSF_AB levels, the assumption of homogeneity of variances was violated (Levene's F(2, 65) = 3.290, p = .044). A Welch's ANOVA was therefore conducted, revealing a statistically significant difference among the three groups, F(2, 32.856) =

24.078, p = 3,6417E-7. Post hoc analysis using the Games-Howell test showed that the control group had significantly different levels compared to both the MCI group (p = 0,000052) and the AD group (p = 0,000004). No significant difference was observed between the MCI and AD groups (p = .051).

- **CSF_pTau**: A Kruskal-Wallis test showed a highly significant difference in CSF_ptau levels among the three groups, $\chi^2(2)$ = 39.417, p = 2,7581E-9. Post hoc analysis with Bonferroni correction revealed that the control group had significantly different levels compared to both the MCI group (p = 4,5787E-7) and the AD group (p = 1,7712E-9). No significant difference was found between the MCI and AD groups (p = .194).
- **Lymphocytes:** A Kruskal-Wallis test showed a statistically significant difference in Lymph levels among the three groups, χ^2 (2) = 15,623, p = 0,000405. Post hoc analysis with Bonferroni correction revealed that the control group had significantly different levels compared to both the MCI group p = 0,049572) and the AD group (p 0,000020). Finally, no significant differences were found between the MCI and AD groups (p = 0,138210).
- **PCR_APC:** A Kruskal-Wallis test showed a highly significant difference in APC_PCR levels among the three groups, χ^2 (2) = 43.188, p = 4,1859E-10 Post hoc analysis with Bonferroni correction revealed that the AD group had significantly different levels compared to both the control group (p = 7,6074E-10) and the MCI group (p = 0,000002). No significant difference was found between the control and MCI groups (p = .054).
- **PCR_Cdh1:** A Kruskal-Wallis test showed a highly significant difference in Cdh1_PCR levels among the three groups, χ^2 (2) = 37.114, p = 8,7247E-9. Post hoc analysis with Bonferroni correction revealed that the AD group had significantly different levels compared to both the control group (p = 2,332E-8) and the MCI group (p = 0,000004). No significant difference was found between the control and MCI groups (p = 0,169).
- **PCR_CDC20:** As the data for CDC20 levels was not normally distributed (Kolmogorov-Smirnov p < .05), a Kruskal-Wallis test was performed. The test showed a highly significant difference among the three groups, χ^2 (2) = 39.963, p < 2,1001E-9. Post hoc analysis with Bonferroni correction revealed that the control group had significantly different levels compared to both the MCI group (p = 0,000002) and the AD group (p = 3,5191E-9) No significant difference was found between the MCI and AD groups (p = .155).

- **PCR_GLUTAMINASA:** As the data for GLUTAMINASA levels was not normally distributed (Kolmogorov-Smirnov $p < .05$), a Kruskal-Wallis test was performed. The test showed a highly significant difference among the three groups, χ^2 (2) = 14.663, p = 0,000655. Post hoc analysis with Bonferroni correction revealed that the control group had significantly different levels compared to AD group (p = 0,000397). No significant difference was found between the MCI and AD groups (p = 0,061) nor among control and MCI group (0.105).
- **CYCLIN_B1:** A Kruskal-Wallis test showed a statistically significant difference in Cyclin_b1 expression levels among the three groups, χ^2 (2) = 10.973, p = 0,004142. Post hoc analysis with Bonferroni correction revealed that the control group had significantly different levels compared to the AD group (p = 0,000930). No other differences were found between the other groups (p=0.171 Control vs MCI; p=0.163 MCI vs AD).
- **Plasma Glutamate:** For plasma_glutamate levels, the assumptions of normality (Kolmogorov-Smirnov $p > .05$) and homogeneity of variances (Levene's $F(2, 65) = 0.634$, $p = .534$) were met. A standard one-way ANOVA was performed and found no statistically significant difference among the three groups, $F(2, 65) = 0.723$, $p = .489$.
- **Plasma Glutamine:** For plasma_glutamine levels, the assumptions of normality (Kolmogorov-Smirnov $p > .05$) and homogeneity of variances (Levene's $F(2, 65) = 2.219$, $p = .117$) were met. A standard one-way ANOVA was performed, revealing a statistically significant difference among the three groups, $F(2, 65) = 11.355$, p = 0,000059. Post hoc analysis using the Bonferroni correction showed that the control group had significantly different levels compared to both the MCI group (p = .010) and the AD group (p = 0,000034). No significant difference was found between the MCI and AD groups (p = .052).
- **CSF_Glutamate:** As the data for CSF_glutamate levels was not normally distributed (Kolmogorov-Smirnov $p < .05$), a Kruskal-Wallis test was performed. The test showed a highly significant difference among the three groups, χ^2 (2) = 20.525, p = 0,000035. Post hoc analysis with Bonferroni correction revealed that the AD group had significantly different levels compared to both the MCI group (p = 0,000019) and the control group (p = .015). No significant difference was found between the MCI and control groups (p = .736).
- **CSF_Glutamine:** For CSF_glutamine levels, the assumptions of normality (Kolmogorov-Smirnov $p > .05$) and homogeneity of variances (Levene's $F(2, 65) = 2.739$, $p = .072$) were met. A standard one-way ANOVA was performed, revealing a statistically significant difference among the three groups, $F(2, 65) = 7.259$, $p = .001$. Post hoc analysis using the Bonferroni correction

showed that the control group had significantly different levels compared to both the MCI group (p = .001) and the AD group (p = .011). No significant difference was found between the MCI and AD groups (p = 1.000).

- **CSF_GABA:** For CSF_GABA levels, the assumption of homogeneity of variances was violated (Levene's F(2, 65) = 24.664, p < .001). A Welch's ANOVA was therefore conducted and found a statistically significant difference among the three groups, F(2, 28.603) = 6.523, p = .005. Post hoc analysis using the Games-Howell test identified that this difference was specifically between the control group and the AD group (p = .010). No significant differences were found between the control and MCI groups (p = .052) or the MCI and AD groups (p = .072).
- **GCLM_PCR:** For GCLM levels, the assumptions of normality (Kolmogorov-Smirnov p > .05) and homogeneity of variances (Levene's F(2, 65) = 2.397, p = .099) were met. A standard one-way ANOVA was performed and found no statistically significant difference among the three groups, F(2, 65) = 1.139, p = .327.
- **GCLC_PCR:** For GCLC levels, the assumptions of normality (Kolmogorov-Smirnov p > .05) and homogeneity of variances (Levene's F(2, 65) = 2.487, p = .091) were met. A standard one-way ANOVA was performed, revealing a statistically significant difference among the three groups, F(2, 65) = 45.492, p = 4,4106E-13. Post hoc analysis using the Bonferroni correction showed that the AD group had significantly different levels compared to both the control group (p = 4,5153E-8) and the MCI group (p = 2,7074E-13). No significant difference was found between the control and MCI groups (p = .265)."
- **GPC_PCR:** For GPX levels, the assumption of homogeneity of variances was violated (Levene's F(2, 65) = 7.798, p = .001). A Welch's ANOVA was therefore conducted and found a highly significant difference among the three groups, F(2, 26.334) = 12.960, p = 0,000121. Post hoc analysis using the Games-Howell test showed that the control group had significantly different levels compared to both the MCI group (p = .001) and the AD group (p = 0,000063). No significant difference was found between the MCI and AD groups (p = .088).
- **AGE:** For AGE levels, the assumption of homogeneity of variances was violated (Levene's F(2, 65) = 5.793, p = .005). A Welch's ANOVA was therefore conducted and found a statistically significant difference among the three groups, F(2, 28.656) = 4.965, p = .014. Post hoc analysis using the Games-Howell test identified that this difference was specifically between the control group and the AD group (p = .010). No significant differences were found between the control and MCI groups (p = .245) or the MCI and AD groups (p = .060).

- **MDA:** As the data for MDA levels was not normally distributed (Kolmogorov-Smirnov $p < .05$), a Kruskal-Wallis test was performed. The test showed a highly significant difference among the three groups, χ^2 (2) = 13,210, p = 0,001. Post hoc analysis with Bonferroni correction revealed that the AD group had significantly different levels compared to the control group (p = 0,000856). No significant difference was found between the MCI and control groups (p = .134), nor between MCI and AD group (p=0.085).
- **AOPP:** For AOPP levels, the assumptions of normality (Kolmogorov-Smirnov $p > .05$) and homogeneity of variances (Levene's F(2, 65) = 1.597, p = .210) were met. A standard one-way ANOVA was performed, revealing a statistically significant difference among the three groups, F(2, 65) = 5.644, p = .005. Post hoc analysis using the Bonferroni correction showed this was driven by a significant difference between the control group and the AD group (p = .004). No significant differences were found between the control and MCI groups (p = .228) or the MCI and AD groups (p = .127).
- **Gsk3b:** As the data for GSK3B levels was not normally distributed (Kolmogorov-Smirnov $p < .05$), a Kruskal-Wallis test was performed. The test showed a highly significant difference among the three groups, χ^2 (2) = 20.059, p = 0,000044. Post hoc analysis with Bonferroni correction revealed that the control group had significantly different levels compared to both the AD group (p = .001) and the MCI group (p = 0,000043). No significant difference was found between the AD and MCI groups (p = 1.000).
- **PKR_PCR:** For PKR levels, the assumption of homogeneity of variances was violated (Levene's F(2, 65) = 4.096, p = .021). A Welch's ANOVA was therefore conducted and found a highly significant difference among the three groups, F(2, 36.796) = 17.550, p = 0,000004. Post hoc analysis using the Games-Howell test showed that the control group had significantly different levels compared to both the MCI group (p = 0,000025) and the AD group (p = .001). No significant difference was found between the MCI and AD groups (p = .674).
- **TFAM:** For TFAM levels, the assumptions of normality (Kolmogorov-Smirnov $p > .05$) and homogeneity of variances (Levene's F(2, 65) = 2.938, p = .060) were met. A standard one-way ANOVA was performed, revealing a statistically significant difference among the three groups, F(2, 65) = 13.946, p = 0,000009. Post hoc analysis using the Bonferroni correction showed that the control group had significantly different levels compared to both the MCI group (p = 0,000010) and the AD group (p = 0,000271). No significant difference was found between the MCI and AD groups (p = 1.000).

TABLE
